# Assessing sequence heterogeneity in Chlorellaceae DNA barcode markers for phylogenetic inference

**DOI:** 10.1186/s43141-023-00550-5

**Published:** 2023-10-18

**Authors:** Ee Bhei Wong, Nurhaida Kamaruddin, Marina Mokhtar, Norjan Yusof, Raja Farhana R. Khairuddin

**Affiliations:** 1 Department of Biology, Faculty of Science and Mathematics, Universiti Pendidikan Sultan Idris, 35900 Tanjong Malim, Perak Malaysia; 2 Centre of Research for Computational Sciences and Informatics for Biology, Bioindustry, Environment, Agriculture, and Healthcare (CRYSTAL), Universiti Malaya, Kuala Lumpur, Malaysia

**Keywords:** Sequence heterogeneity, DNA barcode marker, Chlorellaceae species, Phylogenetic inference

## Abstract

Phylogenetic inference is an important approach that allows the recovery of the evolutionary history and the origin of the Chlorellaceae species. Despite the species’ potential for biofuel feedstock production, their high phenotypic plasticity and similar morphological structures among the species have muddled the taxonomy and identification of the Chlorellaceae species. This study aimed to decipher Chlorellaceae DNA barcode marker heterogeneity by examining the sequence divergence and genomic properties of 18S rRNA, ITS (ITS1-5.8S rRNA-ITS2-28S rRNA), and *rbcL* from 655 orthologous sequences of 64 species across 31 genera in the Chlorellaceae family. The study assessed the distinct evolutionary properties of the DNA markers that may have caused the discordance between individual trees in the phylogenetic inference using the Robinson-Foulds distance and the Shimodaira-Hasegawa test. Our findings suggest that using the supermatrix approach improves the congruency between trees by reducing stochastic error and increasing the confidence of the inferred Chlorellaceae phylogenetic tree. This study also found that the phylogenies inferred through the supermatrix approach might not always be well supported by all markers. The study highlights that assessing sequence heterogeneity prior to the phylogenetic inference could allow the approach to accommodate sequence evolutionary properties and support species identification from the most congruent phylogeny, which can better represent the evolution of Chlorellaceae species.

**Supplementary Information:**

The online version contains supplementary material available at 10.1186/s43141-023-00550-5.

## Introduction

Species phylogeny provides a piece of vital information on the evolutionary history and origin of a species. However, generating an accurate estimation of the true species phylogeny remains a challenge. A few factors, such as choosing which genomic region(s) should be included and which approach is the most appropriate to be used for the selected regions in phylogenetic inference, could affect the reliability of the species phylogeny inferred [1, 2]. The sequence heterogeneity properties may further complicate the process of directly identifying the best approach for inferring evolutionary relationships between species. Each genomic region holds specific evolutionary information about the species that could infer a different phylogenetic tree due to the robustness of the phylogenetic estimation approach or the convoluted evolutionary properties of the genomic regions [2–5].

Accommodating multiple regions of genomic information for phylogenetic inference can be achieved through the supermatrix approach [6–9]. The approach attempts to capture the maximum evolutionary properties by concatenating all orthologous sequences into a supermatrix (super-alignment), which can reduce the stochastic errors in phylogenetic estimations and is more likely to result in a resolved phylogeny. Opinions differ in the approaches, either concatenating genomic sequences or performing separate analyses to construct a reliable species phylogeny [10–13].

A wide range of microalgae applications has received renewed interest, specifically as an essential candidate source of the product in bio-refineries and biofuel feedstock production. In addition, the desirable characteristics of microalgae offer new possibilities for more effective and affordable alternative energy resources [14–17]. Chlorellaceae is one of the most prominent taxonomy families of microalgae, with more than 200 known species across 56 genera, and *Chlorella* is the genus with the highest number of species (14%) among all genera. However, to date, only 32 *Chlorella* species have been identified at the species level, resulting in obscure taxonomic and phylogenetic relationships between microalgae species. Furthermore, their high phenotypic plasticity and similar morphological structure [18, 19] further muddle the process for accurate species identification.

A rigid physiological structure, that is, a thick cell wall, of Chlorellaceae species can hinder the extraction process for obtaining quality genomic DNA [20, 21], which may prompt the preference for using a single marker in the phylogenetic inference for species taxonomy and identification [22–24]. With the availability of universal primers for 18S rRNA, the region has been commonly used as a standard marker to identify and classify Chlorellaceae species [25]. Other molecular markers, such as mitochondrial genes (e.g., *COI*) and chloroplast genes (e.g., *rbcL* and *tufA*) that can also be used for Chlorellaceae species identification are still accessibly limited. Species identification deduced from a single-marker tree can neglect the actual presence of various evolutionary signals in different genomic markers across species [26–30]. Thus, using a single marker may not be adequate to denote the evolutionary relationship between the Chlorellaceae species, and the evolutionary relationship between the species remains obscure.

This study examined the sequence divergence and genomic properties of 18S rRNA gene (18S), ITS region (ITS) that consists of internal transcribed spacer 1, 5.8S rRNA gene, internal transcribed spacer 2 and 28S rRNA gene, and ribulose-1,5-bisphosphate carboxylase large chain gene (*rbcL*) that are commonly used as DNA barcode markers for species identification across 31 Chlorellaceae genera. We inferred a phylogenetic tree for each marker and assessed the congruency among trees. Furthermore, we explored the effect of the supermatrix approach in accommodating the heterogeneous sequences of barcode markers to elucidate the evolutionary relationship between species in the Chlorellaceae family.

## Materials and methods

### Taxon selection and sequence retrieval

Available DNA barcode marker sequences of 18S rRNA, ITS (ITS1-5.8S-ITS2-28S), and *rbcL* of the Chlorellaceae family were retrieved from the National Centre of Biotechnology Information (NCBI) GenBank database. Poor quality and unannotated sequences were filtered using Basic Local Alignment Search Tool (BLAST) with the cutoff *e* value 10^–15^, the percentage of identity (> 40%) and the query coverage (> 40%). Each sequence was mapped to its genome annotation, and redundant sequences were removed prior to orthologous clustering via Proteinortho [31]. Using the tree-based method, we excluded ambiguous sequences of a species that were evidently clustered with other species, which may arise due to misclassification or horizontal gene transfer events. The final dataset consisted of 655 sequences from 64 species across 31 genera of the Chlorellaceae family (Table S1). We also retrieved the sequences of these three markers from species of the Oocystaceae family as an outgroup (Table S1). The GC content of the marker sequences was calculated and compared between the genera. The transition and transversion (Ts/Tv) ratio and the genetic distance between marker sequences were estimated using the Kimura-2-parameter (K2P) model. Homogeneity between sequences was assessed using the disparity index (*I*_*D*_) [32] between and within the genus Chlorellaceae.

### Inferring reference single-marker trees

The filtered sequences of each DNA marker with the outgroup sequences were aligned using a global alignment algorithm (G-INSI) with 1000 iterations using MAFFT v7.4 [33]. Any region with more than 70% gaps in each alignment was trimmed while retaining 80% of the original alignment length. The optimal nucleotide substitution model selection of TIM1 + I + Γ, GTR + I + Γ, and GTR + I + Γ was determined using Modeltest-NG v0.1 [34] with the corrected Akaike information criterion (AICc) for 18S, ITS, and *rbcL,* respectively. The suggested best-fit evolutionary model for each marker was implemented in the RAxML-NG program [35] to infer the maximum likelihood trees with 1000 bootstraps, which were used as reference single-marker trees. Felsenstein bootstrap proportion (FBP) and transfer bootstrap expectation (TBE) bootstraps were used to indicate a node of a high confidence clade of species with more than 70% FBP or TBE bootstrap values.

### Assessing congruency between single-marker trees

To evaluate the congruency of the evolutionary signals between individual trees of the barcode markers in the Chlorellaceae family, a total of 43 sequences were used as a set of representative sequences of 14 Chlorellaceae species from 10 genera. The representative sequences of the 14 species were found to be commonly shared by all of the single-marker trees and were clustered within their species clade with the highest bootstrap support values. The bipartition differences between the single-marker trees were computed using the normalized Robinson-Foulds (nRF) distance [36]. The Shimodaira Hasegawa test (SH test) [37] with a significant *P* value (0.01) was used to assess the congruency between each of the estimated trees and marker sequences through the estimated site log-likelihood matrices.

### Inferring supermatrix trees and accessing congruency between supermatrix trees

To generate supermatrix trees for the 14 species, the sequences of shared taxa among the barcode markers were individually aligned before proceeding with sequence concatenation in pairs and all three markers together. Maximum likelihood trees with 1000 bootstraps were inferred for all supermatrix datasets under the partition evolutionary models that were best estimated for each marker, as previously described for inferring reference single-marker tree. Each inferred supermatrix tree was compared with single-marker trees using the nRF distance. An SH test with a significant *P* value (0.01) was used to assess the congruency of each of the estimated trees to every marker and supermatrix dataset through the estimated site log-likelihood matrices. Phylogenetic trees of all possible marker arrangements of the supermatrix datasets were also constructed using the same parameters and evaluated via the SH test. The information about Chlorellaceae clades, that is, *the Chlorella* clade and *Parachlorella* clade, based on Luo et al. (2010), was mapped onto supermatrix trees and single-marker trees for comparison purposes [38].

## Results and discussion

### Sequence divergence of 18S, ITS, and *rbcL* across Chlorellaceae family

**Fig. 1:**
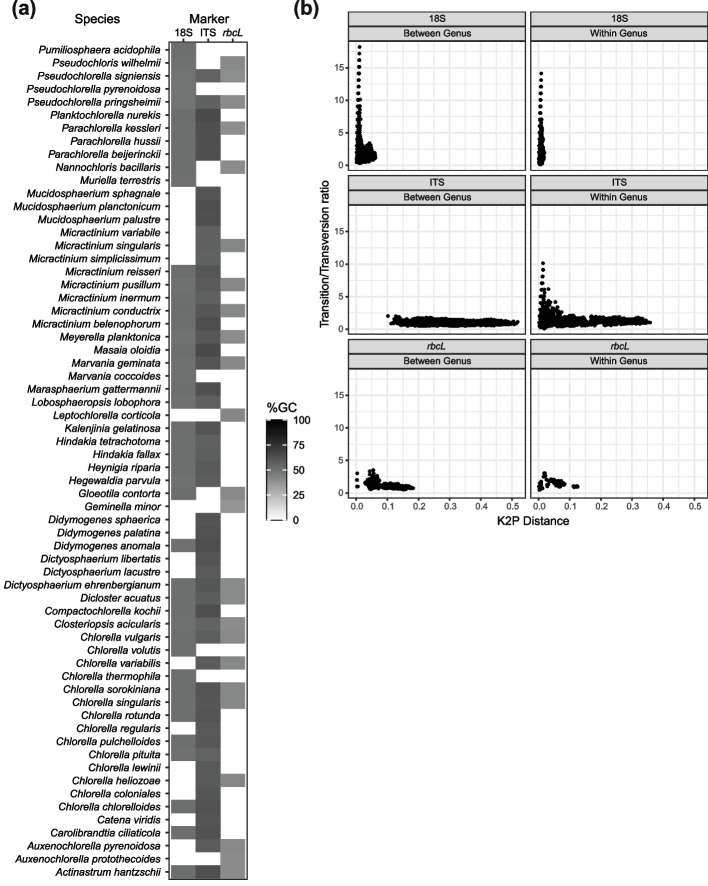
Distribution of 18S rRNA, ITS, and *rbcL* across the Chlorellaceae family. **a** The presence (gray to black) and absence (white) of each marker were mapped according to their %GC content for all 31 Chlorellaceae species; **b** the transition/transversion (Ts/Tv) ratio was mapped against the K2P distance for paired sequences of 18S, ITS, and *rbcL* within and between genera of Chlorellaceae species

Three DNA barcode markers, 18S, ITS, and *rbcL,* of the Chlorellaceae family, with the majority species from the *Chlorella* genus, were retrieved and examined for their compositional bias and genetic features. A total of 655 orthologs from 64 unique species across 31 genera from the Chlorellaceae family were analyzed in this study. Only ~ 21% (14 species) of the 64 species had all three markers available (Fig. 1a). 18S and ITS sequences are present in most Chlorellaceae species, whereas the availability of *rbcL* sequences is limited across species.

The base composition of the DNA markers varied significantly across the species of the Chlorellaceae family. The ITS sequences were GC richer, followed by the 18S and *rbcL* sequences with an average GC percentage of 59.02% ± 0.02, 50.32% ± 0.005, and 40.82% ± 0.01, respectively (Fig. S1). The heterogeneity of GC content in the 18S region was less notable than that in the other two biomolecular marker sequences across species. 18S was also found to have similar GC and AT contents (48.69 to 51.43%) across all genera in the Chlorellaceae family. The *rbcL* sequences have GC content variation ranging from 36.85% in *Geminella minor* to 42.46% in *Micractinium pusillum*, despite being GC-poor.

Genetic distance was estimated using the K2P model and plotted against the Ts/Tv ratio of Chlorellaceae species within and between genera for all markers (Fig. 1b). The Ts/Tv ratio examination shows a decrease with increasing genetic distance for all three markers among the species. Greater genetic distance dispersion among species between genera was detected in ITS compared to *rbcL* and 18S genes. *Chlorella* species showed the highest significant distance (0.3583), contributing to the dispersion of genetic distance in the ITS. The Ts/Tv ratio revealed that high transition rates in ITS and 18S markers were mainly contributed (> 90%) by species in the *Chlorella* genus compared with other species. In addition, a high genetic distance in ITS is commonly observed among species under the *genus Auxenochlorella*.

We evaluated the heterogeneity of the sequences within the Chlorellaceae genus using the *I*_*D*_ for each marker (Table S2). The majority of the sequences were homogenous within their genus, with no significant difference at a *P* value of 0.05. However, *Chlorella* species were found to have the highest frequency of significant heterogeneity among their sequences in all DNA barcode markers: 18S (5.20%), ITS (27.17%), and *rbcL* (23.30%). Significant heterogeneity between the 18S rRNA and ITS sequences was also detected within the *genera Dictyosphaerium and Micractinium*.

Sequence heterogeneity comparisons between the Chlorellaceae genera showed that the ITS sequences had the highest frequency of significant heterogeneity, with the majority of genera having a frequency range of 20 to 68%. More than 80% *I*_*D*_ of pairwise comparisons in *Planktochlorella* and *Masaia* indicate that both genera have the most divergent nucleotide sequences compared with sequences from other genera. Low heterogeneity was detected between genera for most 18S and *rbcL* sequences, ranging from 0.5 to 10% and from 5 to 25%, respectively.

*Pseudochlorella* species showed a high frequency (> 80%) of heterogeneous sequence composition in the 18S region. All *rbcL* sequences in the *Geminella* genus and more than 90% of the *Parachlorella* sequence had the greatest percentage of significant heterogeneity among other genus sequences. This finding suggests that the higher sequence heterogeneity between the Chlorellaceae genus than within the genus indicates that sequences from the same species are more likely to be clustered together in a clade of an estimated phylogenetic tree. However, the variation between individual markers can affect the inference of the Chlorellaceae phylogenetic tree. The information obtained on sequence heterogeneity across Chlorellaceae barcode markers supports the allocation of an appropriate evolutionary model specific to each marker in phylogenetic inference [39, 40].

#### Discordance between trees of Chlorellaceae DNA barcode markers

Maximum likelihood trees were inferred from 18S, ITS, and *rbcL *sequences using their best-estimated nucleotide substitution models. The single-marker trees were examined for congruency and evolutionary divergence. Each marker tree comprised 43 sequences of 14 Chlorellaceae species from 10 genera, of which the majority were classified under the *Chlorella* and *Parachlorella* clades, followed by two species under the *Pseudochlorella* genus and one *Marvania* species.

**Fig. 2:**
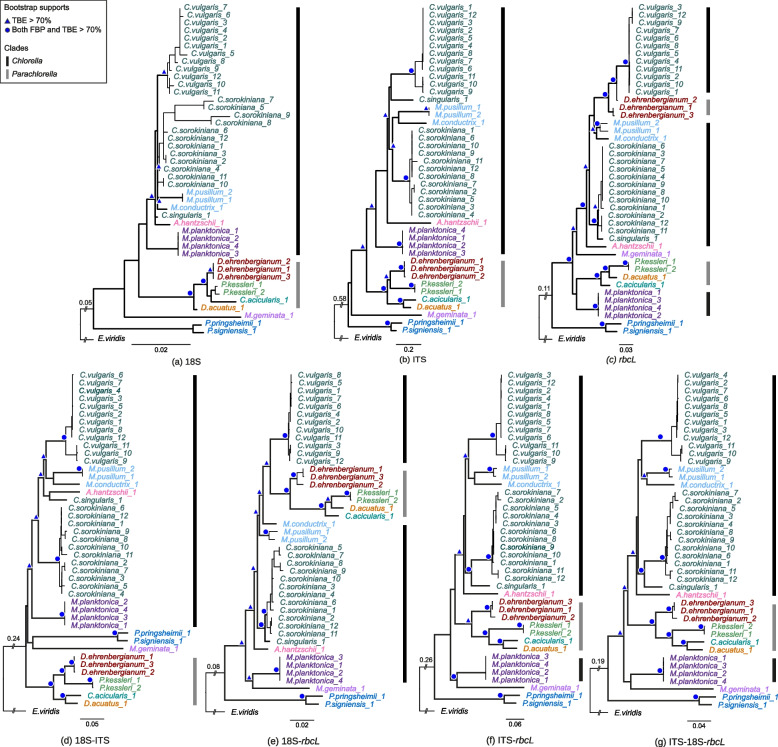
Maximum likelihood (ML) trees for single-marker and supermatrix of Chlorellaceae species. **a**–**c** 18S, ITS, and *rbcL* of 14 Chlorellaceae pruned from each reference single-marker tree (File S1). **d**–**g** Maximum likelihood phylogenetic trees of supermatrix markers 18S-ITS, 18S-*rbcL*, ITS-*rbcL*, and 18S-ITS-*rbcL*. All species were colored according to their genus, and the trees were rooted in an outgroup, *Eremosphaera viridis*. The thick internal branches depict the phylogeny of the relationship between the Chlorellaceae species. The branch length is indicated by the number of substitutions per site. Felsenstein’s bootstrap proportion (FBP) and transfer bootstrap expectation (TBE) bootstrap values ≥ 70% are shown at the corresponding nodes with either a blue triangle (FBP) or blue circle (FBP and TBE). The species classified under the Chlorella clade (dark gray) and Parachlorella clade (light gray) are marked accordingly in the ML trees. The two forward slashes indicate the trimmed branches with an actual branch length above the corresponding branches

Distinct evolutionary signals of each marker generated different phylogenetic trees for the Chlorellaceae species (Fig. 2). The single-marker trees had more than 60% bipartition differences (Table 1 (part a)), which mainly involved the leaf nodes at the tip of the tree, illustrating the conflicting relationships between the Chlorellaceae species. The sequences of the same species were clustered together and shared an ancestor across the three markers, with high bootstrap support (> 70%). High incongruency between single-marker trees was found within the species clades that depicted significant intraspecific sequence variations, although sequences of the same species tended to be clustered together.

**Table 1:** Congruency assessment of single-marker and supermatrix trees using Robinson Foulds (nRF) distance (%)  and SH test (*P* value)

a)	Tree	Single-marker tree			Supermatrix tree			
		18S	ITS	*rbcL*	18S-ITS	18S-*rbcL*	ITS-*rbcL*	ITS-18S-*rbcL*
		nRF (%)				nRF (%)		
Single-marker tree	18S	0	62.5	77.5	50	70	72.5	65
	ITS	62.5	0	80	47.5	75	45	50
	*rbcL*	77.5	80	0	77.5	55	72.5	70
Supermatrix tree	18S-ITS	50	47.5	77.5	0	70	52.5	45
	18S-*rbcL*	70	75	55	70	0	62.5	47.5
	ITS-*rbcL*	72.5	45	72.5	52.5	62.5	0	25
	ITS-18S-*rbcL*	65	50	70	45	47.5	25	0
b)	Marker dataset	Single-marker tree			Supermatrix tree			
		18S	ITS	*rbcL*	18S-ITS	18S-*rbcL*	ITS-*rbcL*	ITS-18S-*rbcL*
		*P* value				*P *value		
Single-marker	18S	**0.998**	0.001	0	**0.215**	0.003	0	0.008
	ITS	0	**0.827**	0	**0.914**	0	**0.466**	**0.466**
	*rbcL*	0	0	**0.999**	0	**0.1**	0	0
Supermatrix	18S-ITS	0	**0.259**	0	**1**	0	**0.179**	**0.324**
	18S-*rbcL*	2×10–04	4×10–05	**0.031**	1×10–04	**1**	0.001	**0.01**
	ITS-*rbcL*	0	**0.164**	0	**0.021**	0	**0.99**	**0.838**
	ITS-18S-*rbcL*	0	**0.14**	0	**0.085**	0	**0.724**	**0.996**
c)	Marker dataset	Collapsed single-marker tree			Collapsed supermatrix tree			
		18S	ITS	*rbcL*	18S-ITS	18S-*rbcL*	ITS-*rbcL*	ITS-18S-*rbcL*
		*P* value				*P* value		
Single-marker	18S	**0.957**	**0.308**	0	**0.365**	**0.213**	**0.233**	**0.233**
	ITS	0	**0.579**	0	**0.986**	0	**0.308**	**0.309**
	*rbcL*	0	0	**0.999**	0	**0.05**	**0.015**	**0.015**
Supermatrix	18S-ITS	**0.024**	**0.648**	0	**0.987**	0.001	**0.499**	**0.499**
	18S-*rbcL*	0.006	0.007	**0.114**	0.008	**0.864**	**0.906**	**0.906**
	ITS-*rbcL*	0	**0.055**	**0.23**	**0.097**	**0.045**	**0.949**	**0.949**
	ITS-18S-*rbcL*	0.001	**0.086**	1×10–04	**0.131**	**0.048**	**0.959**	**0.948**

*Footnote*: a) Bipartition differences between the compared trees (%nRF). The SH test *P* value was calculated to assess the congruency between b) the single-marker and the supermatrix trees, and c) the collapsed trees, to the marker datasets. The *P* values > 0.01 are in bold

Despite the incongruency of tree topology within species, ITS and 18S marker trees have a more similar evolutionary history between the Chlorellaceae species, with 37% identical tree topology to the *rbcL* marker tree (< 23%). The clustering of species in the *Parachlorella* clade in the marker trees is likely to cause conflicts between tree topologies. The 18S marker tree shared a topology similar to that of the *rbcL* marker tree with either *Closteriopsis acicularis* or *Dicloster acuatus* derived individually from the *Parachlorella* clade ancestor. The *rbcL* sequences of another *Chlorella* species, *Meyerella planktonica*, were inferred to share a common ancestor with other species in the *Parachlorella* clade. *Dictyosphaerium ehrenbergianum* shared a common ancestor with *Chlorella vulgaris* and clustered together with other *Chlorella* species in the *rbcL* marker tree, in contrast to the ITS and 18S marker trees.

We further evaluated the tree congruency of the single-marker datasets using the SH test at *P* value < 0.01. The single-marker trees were strongly incongruent in the analysis, and none of the single-marker trees was supported by any of the other markers (Table 1 (part b)). Although other single-marker trees have been rejected, most supermatrix datasets support the ITS tree. We collapsed the taxa of the same species to reduce tree topology conflicts within the species clades and conducted an SH test. Only the 18S sequences supported the Chlorellaceae species lineages depicted by the ITS marker tree, whereas the rest of the sequences rejected other marker tree hypotheses, suggesting incongruent evolutionary signals between the markers (Table 1 (part c)).

The conflicting signals imposed through these marker trees indicate heterogeneous evolutionary events, such as recombinant, coalescent, and horizontal gene transfer, which have affected the microalgae lineages at the level of the organism [30]. The discordance between individual marker trees caused by sequence heterogeneity properties could complicate the inference of the evolutionary relationship between Chlorellaceae species. This suggests that using a single marker to infer the phylogeny of Chlorellaceae can distort the evolutionary relationship between these species and further introduce ambiguity in species assignment.

#### The effect of the supermatrix approach in accommodating the sequence heterogeneity for the Chlorellaceae species phylogeny

Adding more information to the phylogenetic analysis requires careful assessment of the selected genomic regions and appropriate phylogenetic approaches, especially when accommodating heterogeneous evolutionary signals across Chlorellaceae DNA markers. We explored the effect of the supermatrix approach by concatenating these markers pairwise and all the markers together to infer supermatrix trees. Our findings showed that none of the Chlorellaceae supermatrix trees had the same tree topology as any single-marker tree (Fig. 2), with an nRF bipartition difference of up to 77.5% (Table 1 (part a)). We tested whether the trees were a good representation of the evolutionary relationship between Chlorellaceae species. The supermatrix trees had a better fit than the single-marker trees to represent the evolution of Chlorellaceae species, in which the topologies are likely to be restricted by a specific marker (Table 1 (part b)). The* rbcL* marker rejected most of the supermatrix trees, but the 18S-*rbcL* tree rejected the majority because of the distinct species clustering in the *Parachlorella* clade, in which *D. acuatus* and *C. acicularis* clustered together with *P. kessleri* in both trees. We also tested all possible marker arrangements for each concatenation of the supermatrix datasets, which showed no significant differences between the inferred trees (Table S3). The results suggest significant congruency of the inferred trees to represent the evolution of Chlorellaceae species from the supermatrix marker datasets despite the differences between marker arrangements and sequence heterogeneity.

A comparison between the supermatrix tree topologies revealed that sequences from the same species were consistently clustered together, and *Pseudochlorella* species were found at the most ancestral node of the Chlorellaceae family (Fig. 2e–f). The nRF bipartition distances among the supermatrix trees (Table 1 (part a)) significantly decreased with an increase in marker number, but were not affected by the supermatrix sequence length. Fewer conflicts found between the supermatrix trees are also likely due to low intraspecific topological variations within each species clade in the supermatrix trees compared to the single-marker trees. Thus*,* the congruency between supermatrix trees increased when the taxa within each species clade collapsed into a single species representation (Table 1 (part c)).

The pairwise supermatrix trees distinctly positioned the *Parachlorella* clade on the trees, contributing to incongruence (Fig. 2d–f). However, the clustering of *D. ehrenbergianum* with *P. kessleri*, *C. acicularis,* and *D. acuatus* in the Parachlorella clade was consistent across these supermatrix trees with high bootstrap support (> 70% FBP and TBE). *D. ehrenbergianum* is an unresolved nomenclature, which was found to be clustered with the *Chlorella* [41] and *Parachlorella* species [42]. A few species, that is, *M. pusillum*, *M. planktonica*, and *M. geminata*, also showed differing positions in these pairwise supermatrix trees. The 18S-ITS supermatrix tree alone did not rule out *M. planktonica* species from the Chlorella clade to be clustered with *M. geminata*. Both species (i.e., *M. planktonica* and *M. geminata*) are morphologically similar in the absence of a pyrenoid in their chloroplasts [43].

The concatenation of the individual Chlorellaceae DNA barcode markers improved the congruency between the phylogenetic trees. The ITS-18S-*rbcL* supermatrix tree showed the best agreement in representing the evolution of the species in the Chlorellaceae family based on the three DNA markers used in this study. The collapsed ITS-*rbcL* tree shares a similar species phylogeny topology to the ITS-18S-*rbcL* tree and is also supported by all single markers and supermatrix datasets as the conflicts within the species clades are resolved.

## Conclusion

This study examined the sequence heterogeneity of the DNA barcode markers 18S, ITS, and *rbcL*, which are commonly used for phylogenetic analysis and species assignment of the Chlorellaceae family. We found that each marker of the Chlorellaceae family had distinct evolutionary properties, with each marker tree depicting incongruent evolutionary relationships between Chlorellaceae species evidently at the genus level. Thus, information from a single marker may not be adequate for inferring the phylogeny of Chlorellaceae species or as a reference for identifying Chlorellaceae species taxonomy.

The study has also preliminarily demonstrated that the supermatrix approach could resolve the conflicts between single-marker trees of the Chlorellaceae species. Through the supermatrix approach, the concatenation of the Chlorellaceae DNA barcoding markers reduces the stochastic error and increases the confidence of the inferred phylogeny. Nonetheless, the findings of this study are only based on a few commonly used markers of the Chorellaceae species, which must be interpreted with caution are highly subjected to the availability of the sequence markers.

Concatenating all available sequences indiscriminately in the supermatrix approach may interfere with the underlying phylogenetic signals within each gene, resulting in the supermatrix phylogeny may not always be supported by all markers. Therefore, a careful assessment is suggested of the sequence characteristics of the DNA barcode markers, which could be embedded in the supermatrix construction for accommodating distinct evolutionary markers properties. This could further improve the phylogenetic inference for obtaining a reliable species phylogeny that could better represent the evolution of the Chlorellaceae species.

### Supplementary Information

**Additional file 1: Fig. S1.** Distribution of GC content (%) of 18S, ITS and *rbcL* across the Chlorellaceae genus. The horizontal lines represent the mean of GC content (%) across each marker; ITS (59.02%), 18S (50.32%), and *rbcL* (40.82%).

**Additional file 2: Table S1.** Accession numbers and GC content (%) of 18S, ITS and *rbcL* marker with the outgroups. **Table S2.** K2P genetic distance and disparity index (*I*_*D*_) between and within the Chlorellaceae genus. **Table S3.** Normalised Robinson Foulds(nRF) and Shimodaira Hasegawa test(SH test) on supermatrix marker datasets with alternative marker arrangements.

## Data Availability

All data generated or analyzed during this study are included in this published article and its supplementary information files.

## Abbreviations

DNA: Deoxyribonucleic acid
*rbcL*: Ribulose-1,5-bisphosphate carboxylase large chain
rRNA: Ribosomal ribonucleic acid
ITS: Internal transcribed spacer
GC: Guanine and Cytosine
Ts/Tv: Transition/transversion
K2P: Kimura-2-parameter
*I*_*D*_: Disparity Index
FBP: Felsenstein bootstrap proportion
TBE: Transfer bootstrap expectation
nRF: Normalized Robinson-Foulds
SH test: Shimodaira Hasegawa test
