# Supplementary material for: Assessing sequence heterogeneity in Chlorellaceae DNA barcode markers for phylogenetic inference
Source: J Genet Eng Biotechnol. 2023 Oct 18;21:104. doi: 10.1186/s43141-023-00550-5 (PMC10584744; doi:10.1186/s43141-023-00550-5)

Fig. S1. Distribution of GC content (%) of 18S, ITS and *rbcL* across the Chlorellaceae genus. The horizontal lines represent the mean of GC content (%) across each marker; ITS (59.02%), 18S (50.32%), and *rbcL* (40.82%).

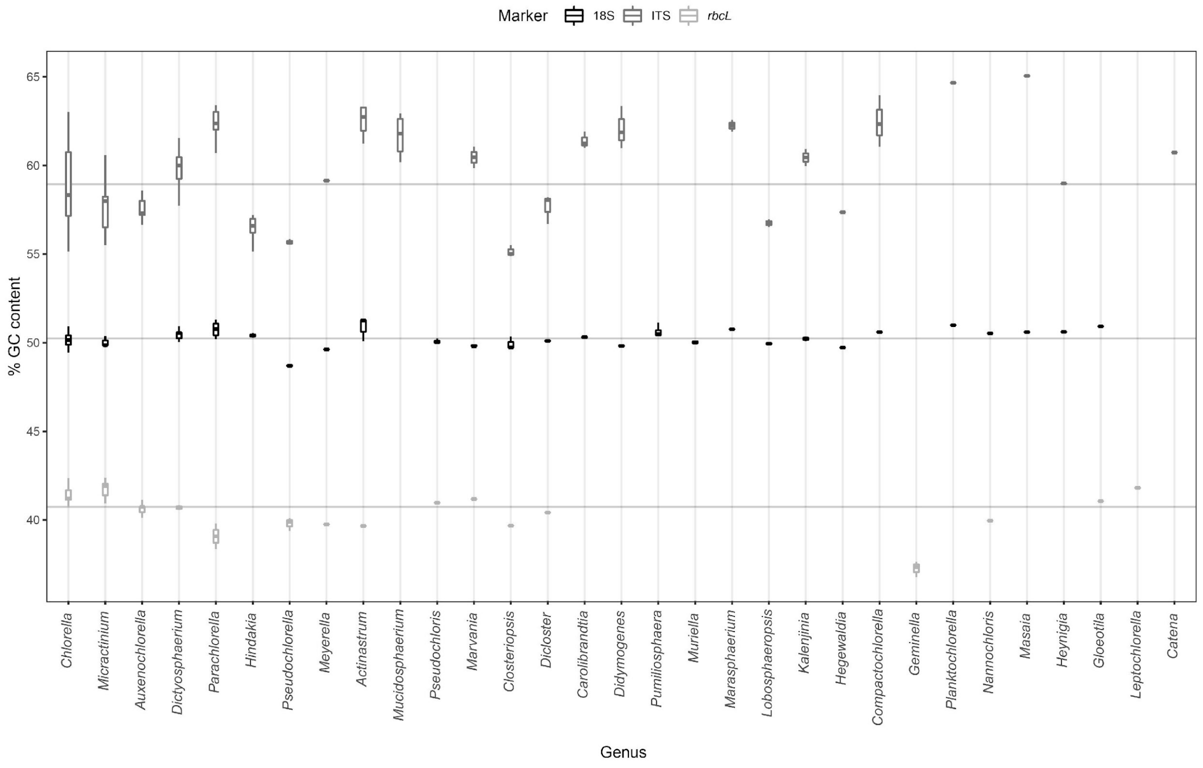

Supplement: Supplementary file 1 — Additional file 1: Fig. S1. Distribution of GC content (%) of 18S, ITS and rbcL across the Chlorellaceae genus. The horizontal lines represent the mean of GC content (%) across each marker; ITS (59.02%), 18S (50.32%), and rbcL (40.82%). [file 43141_2023_550_MOESM1_ESM.pdf]
