# Supplementary material for: Assessing sequence heterogeneity in Chlorellaceae DNA barcode markers for phylogenetic inference
Source: J Genet Eng Biotechnol. 2023 Oct 18;21:104. doi: 10.1186/s43141-023-00550-5 (PMC10584744; doi:10.1186/s43141-023-00550-5)
Supplement: Supplementary file 2 — Additional file 2: Table S1. Accession numbers and GC content (%) of 18S, ITS and rbcL marker with the outgroups. Table S2. K2P genetic distance and disparity index (ID) between and within the Chlorellaceae genus. Table S3. Normalised Robinson Foulds(nRF) and Shimodaira Hasegawa test(SH test) on supermatrix marker datasets with alternative marker arrangements. [file 43141_2023_550_MOESM2_ESM.zip › Amended_Table_S2R1.docx]

| Table S2. K2P genetic distance and disparity index (*I_D_*) between and within the Chlorellaceae genus. | | | | | | | | | | | |
| --- | --- | --- | --- | --- | --- | --- | --- | --- | --- | --- | --- |
| Marker | Genus | Within Genus | | | | | Between Genus | | | | |
|  |  | K2P Genetic Distance | | | | Disparity Index (*I_D_*) | K2P Genetic Distance | | | | Disparity Index (*I_D_*) |
|  |  | N | Min | Mean | Max | % | N | Min | Mean | Max | % |
| 18S | *Actinastrum* | 15 | 0.0000 | 0.0014 ± 0.0014 | 0.0043 | 0.00% (0) | 1440 | 0.0011 | 0.0131 ± 0.00983 | 0.0470 | 4.93% (71) |
| 18S | *Carolibrandtia* | 3 | 0.0005 | 0.0009 ± 0.0004 | 0.0014 | 0.00% (0) | 729 | 0.0055 | 0.0166 ± 0.01021 | 0.0470 | 2.47% (18) |
| 18S | *Chlorella* | 4465 | 0.0000 | 0.0045 ± 0.0033 | 0.0190 | 5.20% (232) | 14345 | 0.0006 | 0.0191 ± 0.01013 | 0.0509 | 5.74% (824) |
| 18S | *Closteriopsis* | 3 | 0.0000 | 0.0000 | 0.0000 | 0.00% (0) | 729 | 0.0069 | 0.0255 ± 0.00979 | 0.0531 | 17.56% (128) |
| 18S | *Compactochlorella* | 0 | NA | NA | NA | NA | 245 | 0.0011 | 0.0221 ± 0.01057 | 0.0513 | 9.39% (23) |
| 18S | *Dicloster* | 1 | 0.0000 | 0.0000 | 0.0000 | 0.00% (0) | 488 | 0.0028 | 0.0201 ± 0.00837 | 0.0476 | 7.58% (37) |
| 18S | *Dictyosphaerium* | 120 | 0.0000 | 0.0027 ± 0.0036 | 0.0128 | 3.33% (4) | 3680 | 0.0017 | 0.0241 ± 0.00989 | 0.0615 | 5.73% (211) |
| 18S | *Didymogenes* | 1 | 0.0000 | 0.0000 | 0.0000 | 0.00% (0) | 488 | 0.0062 | 0.0161 ± 0.00839 | 0.0399 | 3.28% (16) |
| 18S | *Gloeotila* | 0 | NA | NA | NA | NA | 245 | 0.0079 | 0.0262 ± 0.00568 | 0.0466 | 10.61% (26) |
| 18S | *Hegewaldia* | 1 | 0.0000 | 0.0000 | 0.0000 | 0.00% (0) | 488 | 0.0111 | 0.0199 ± 0.00645 | 0.0407 | 2.05% (10) |
| 18S | *Heynigia* | 0 | NA | NA | NA | NA | 245 | 0.0006 | 0.0118 ± 0.00995 | 0.0413 | 6.53% (16) |
| 18S | *Hindakia* | 190 | 0.0000 | 0.0030 ± 0.0032 | 0.0123 | 0.00% (0) | 4520 | 0.0029 | 0.0165 ± 0.01014 | 0.0538 | 2.68% (121) |
| 18S | *Kalenjinia* | 1 | 0.0000 | 0.0000 | 0.0000 | 0.00% (0) | 488 | 0.0028 | 0.0209 ± 0.00870 | 0.0488 | 4.51% (22) |
| 18S | *Lobosphaeropsis* | 1 | 0.0017 | 0.0017 | 0.0017 | 0.00% (0) | 488 | 0.0031 | 0.0145 ± 0.01000 | 0.0438 | 4.71% (23) |
| 18S | *Marasphaerium* | 1 | 0.0000 | 0.0000 | 0.0000 | 0.00% (0) | 488 | 0.0074 | 0.0273 ± 0.00996 | 0.0526 | 27.87% (136) |
| 18S | *Marvania* | 6 | 0.0023 | 0.0039 ± 0.0017 | 0.0073 | 0.00% (0) | 968 | 0.0222 | 0.0349 ± 0.00717 | 0.0607 | 0.52% (5) |
| 18S | *Masaia* | 0 | NA | NA | NA | NA | 245 | 0.0011 | 0.0213 ± 0.01066 | 0.0512 | 5.71% (14) |
| 18S | *Meyerella* | 10 | 0.0000 | 0.0000 | 0.0000 | 0.00% (0) | 1205 | 0.0101 | 0.0191 ± 0.00778 | 0.0427 | 25.98% (313) |
| 18S | *Micractinium* | 378 | 0.0000 | 0.0062 ± 0.0042 | 0.0143 | 5.56% (21) | 6104 | 0.0011 | 0.0163 ± 0.00982 | 0.0484 | 5.05% (308) |
| 18S | *Muriella* | 6 | 0.0000 | 0.0003 ± 0.0003 | 0.0006 | 0.00% (0) | 968 | 0.0155 | 0.0232 ± 0.00500 | 0.0434 | 2.58% (25) |
| 18S | *Nannochloris* | 0 | NA | NA | NA | NA | 245 | 0.0116 | 0.0253 ± 0.00622 | 0.0439 | 4.49% (11) |
| 18S | *Parachlorella* | 351 | 0.0000 | 0.0028 ± 0.0024 | 0.0102 | 0.57% (2) | 5913 | 0.0023 | 0.0259 ± 0.00948 | 0.0621 | 6.90% (408) |
| 18S | *Planktochlorella* | 0 | NA | NA | NA | NA | 245 | 0.0047 | 0.0257 ± 0.01104 | 0.0553 | 4.08% (10) |
| 18S | *Pseudochlorella* | 21 | 0.0000 | 0.0037 ± 0.0024 | 0.0062 | 0.00% (0) | 1673 | 0.0300 | 0.0444 ± 0.00534 | 0.0621 | 81.05% (1356) |
| 18S | *Pseudochloris* | 15 | 0.0000 | 0.0002 ± 0.0003 | 0.0006 | 0.00% (0) | 1440 | 0.0204 | 0.0287 ± 0.00471 | 0.0441 | 2.85% (41) |
| 18S | *Pumiliosphaera* | 6 | 0.0000 | 0.0048 ± 0.0045 | 0.0097 | 0.00% (0) | 968 | 0.0079 | 0.0261 ± 0.00554 | 0.0454 | 6.30% (61) |
| ITS | *Actinastrum* | 6 | 0.0055 | 0.0668 ± 0.0339 | 0.1020 | 0.00% (0) | 1264 | 0.1769 | 0.2995 ± 0.04392 | 0.4410 | 53.56% (677) |
| ITS | *Auxenochlorella* | 210 | 0.0000 | 0.0032 ± 0.0041 | 0.0216 | 1.43% (3) | 6279 | 0.1380 | 0.2892 ± 0.05228 | 0.4554 | 37.00% (2323) |
| ITS | *Carolibrandtia* | 3 | 0.0000 | 0.0054 ± 0.0038 | 0.0084 | 0.00% (0) | 951 | 0.1289 | 0.2513 ± 0.04587 | 0.4438 | 56.78% (540) |
| ITS | *Catena* | 0 | NA | NA | NA | NA | 319 | 0.3293 | 0.4469 ± 0.02575 | 0.4892 | 27.59% (88) |
| ITS | *Chlorella* | 12561 | 0.0000 | 0.1925 ± 0.1226 | 0.3583 | 27.17% (3413) | 25599 | 0.1182 | 0.3134 ± 0.06024 | 0.5143 | 34.91% (8937) |
| ITS | *Closteriopsis* | 3 | 0.0094 | 0.0284 ± 0.0136 | 0.0402 | 0.00% (0) | 951 | 0.1019 | 0.3077 ± 0.05678 | 0.4684 | 49.21% (468) |
| ITS | *Compactochlorella* | 3 | 0.0380 | 0.0433 ± 0.0071 | 0.0533 | 0.00% (0) | 951 | 0.1288 | 0.3012 ± 0.06618 | 0.4549 | 65.72% (625) |
| ITS | *Dicloster* | 3 | 0.0000 | 0.0032 ± 0.0023 | 0.0048 | 0.00% (0) | 951 | 0.1019 | 0.3133 ± 0.06249 | 0.5185 | 22.92% (218) |
| ITS | *Dictyosphaerium* | 325 | 0.0000 | 0.0764 ± 0.0504 | 0.1737 | 1.23% (4) | 7644 | 0.1122 | 0.3142 ± 0.05903 | 0.4866 | 31.38% (2399) |
| ITS | *Didymogenes* | 3 | 0.0831 | 0.0929 ± 0.0083 | 0.1033 | 0.00% (0) | 951 | 0.1182 | 0.2355 ± 0.04961 | 0.4408 | 51.42% (489) |
| ITS | *Hegewaldia* | 1 | 0.0000 | 0.0000 | 0.0000 | 0.00% (0) | 636 | 0.1889 | 0.2757 ± 0.03498 | 0.4241 | 35.22% (224) |
| ITS | *Heynigia* | 0 | NA | NA | NA | NA | 319 | 0.1707 | 0.2846 ± 0.05303 | 0.4718 | 20.06% (64) |
| ITS | *Hindakia* | 190 | 0.0000 | 0.0293 ± 0.0141 | 0.0541 | 4.74% (9) | 6000 | 0.1376 | 0.2739 ± 0.04821 | 0.4915 | 40.60% (2436) |
| ITS | *Kalenjinia* | 1 | 0.0061 | 0.0061 | 0.0061 | 100.00% (1) | 636 | 0.1288 | 0.3014 ± 0.06621 | 0.4574 | 44.03% (280) |
| ITS | *Lobosphaeropsis* | 1 | 0.0044 | 0.0044 | 0.0044 | 0.00% (0) | 636 | 0.1971 | 0.2804 ± 0.05728 | 0.4844 | 39.78% (253) |
| ITS | *Marasphaerium* | 1 | 0.0263 | 0.0263 | 0.0263 | 0.00% (0) | 636 | 0.1540 | 0.3094 ± 0.06462 | 0.4636 | 62.58% (398) |
| ITS | *Marvania* | 1 | 0.1202 | 0.1202 | 0.1202 | 0.00% (0) | 636 | 0.3186 | 0.4247 ± 0.03728 | 0.5143 | 61.64% (392) |
| ITS | *Masaia* | 0 | NA | NA | NA | NA | 319 | 0.1432 | 0.3109 ± 0.06470 | 0.4741 | 82.45% (263) |
| ITS | *Meyerella* | 6 | 0.0000 | 0.0000 | 0.0000 | 0.00% (0) | 1264 | 0.2027 | 0.2884 ± 0.04089 | 0.4204 | 14.40% (182) |
| ITS | *Micractinium* | 465 | 0.0000 | 0.1652 ± 0.0955 | 0.2839 | 18.49% (86) | 8959 | 0.1380 | 0.2894 ± 0.04496 | 0.4665 | 41.13% (3685) |
| ITS | *Mucidosphaerium* | 28 | 0.0014 | 0.1257 ± 0.0782 | 0.2525 | 3.57% (1) | 2496 | 0.1626 | 0.3450 ± 0.06165 | 0.4967 | 55.89% (1395) |
| ITS | *Parachlorella* | 105 | 0.0000 | 0.0332 ± 0.0413 | 0.1065 | 0.00% (0) | 4575 | 0.1122 | 0.3424 ± 0.06826 | 0.5020 | 67.93% (3108) |
| ITS | *Planktochlorella* | 0 | NA | NA | NA | NA | 319 | 0.1432 | 0.2974 ± 0.06924 | 0.4495 | 80.25% (256) |
| ITS | *Pseudochlorella* | 3 | 0.0076 | 0.0667 ± 0.0419 | 0.0999 | 0.00% (0) | 951 | 0.2901 | 0.4074 ± 0.03909 | 0.5185 | 46.90% (446) |
| *rbcL* | *Actinastrum* | 0 | NA |  | NA | NA | 88 | 0.0525 | 0.0809 ± 0.02895 | 0.1601 | 18.18% (16) |
| *rbcL* | *Auxenochlorella* | 351 | 0.0000 | 0.0442 ± 0.0572 | 0.1256 | 0.00% (0) | 1674 | 0.0364 | 0.0850 ± 0.03787 | 0.1619 | 21.51% (360) |
| *rbcL* | *Chlorella* | 528 | 0.0000 | 0.0478 ± 0.0293 | 0.0833 | 23.30% (123) | 1848 | 0.0026 | 0.0857 ± 0.03676 | 0.1688 | 24.46% (452) |
| *rbcL* | *Closteriopsis* | 0 | NA | NA | NA | NA | 88 | 0.0463 | 0.1073 ± 0.01828 | 0.1562 | 7.95% (7) |
| *rbcL* | *Dicloster* | 0 | NA | NA | NA | NA | 88 | 0.0463 | 0.1080 ± 0.01695 | 0.1530 | 21.59% (19) |
| *rbcL* | *Dictyosphaerium* | 6 | 0.0026 | 0.0319 ± 0.0171 | 0.0529 | 0.00% (0) | 340 | 0.0026 | 0.0750 ± 0.03594 | 0.1660 | 15.59% (53) |
| *rbcL* | *Geminella* | 3 | 0.0019 | 0.0039 ± 0.0015 | 0.0056 | 0.00% (0) | 258 | 0.1268 | 0.1496 ± 0.00869 | 0.1831 | 100.00% (258) |
| *rbcL* | *Gloeotila* | 0 | NA | NA | NA | NA | 88 | 0.0648 | 0.1032 ± 0.02189 | 0.1647 | 20.45% (18) |
| *rbcL* | *Leptochlorella* | 0 | NA | NA | NA | NA | 88 | 0.1435 | 0.1546 ± 0.00552 | 0.1740 | 6.82% (6) |
| *rbcL* | *Marvania* | 0 | NA | NA | NA | NA | 88 | 0.0589 | 0.1071 ± 0.01871 | 0.1761 | 9.09% (8) |
| *rbcL* | *Meyerella* | 6 | 0.0000 | 0.0000 | 0.0000 | 0.00% (0) | 340 | 0.0890 | 0.1055 ± 0.02048 | 0.1739 | 5.88% (20) |
| *rbcL* | *Micractinium* | 10 | 0.0316 | 0.0427 ± 0.0067 | 0.0519 | 0.00% (0) | 420 | 0.0364 | 0.0746 ± 0.03641 | 0.1708 | 12.62% (53) |
| *rbcL* | *Nannochloris* | 0 | NA | NA | NA | NA | 88 | 0.0589 | 0.1098 ± 0.02202 | 0.1831 | 13.64% (12) |
| *rbcL* | *Parachlorella* | 1 | 0.0000 | 0.0000 | 0.0000 | 0.00% (0) | 174 | 0.0464 | 0.1092 ± 0.01539 | 0.1608 | 90.23% (157) |
| *rbcL* | *Pseudochlorella* | 3 | 0.0000 | 0.0328 ± 0.0232 | 0.0496 | 0.00% (0) | 258 | 0.1330 | 0.1500 ± 0.00817 | 0.1716 | 14.73% (38) |
| *rbcL* | *Pseudochloris* | 0 | NA | NA | NA | NA | 88 | 0.1105 | 0.1248 ± 0.01080 | 0.1740 | 7.95% (7) |

Footnote: The number of sequence comparisons (N), minimum (min), average (mean) and maximum (max) K2P distance within and between each Chlorellaceae genus was displayed for 18S, ITS and *rbcL* markers. The disparity index (*I_D_*) shows in percentage with the total number of heterogeneous sequence comparisons (in brackets) within and between the Chlorellaceae genus.
