# Supplementary material for: Assessing sequence heterogeneity in Chlorellaceae DNA barcode markers for phylogenetic inference
Source: J Genet Eng Biotechnol. 2023 Oct 18;21:104. doi: 10.1186/s43141-023-00550-5 (PMC10584744; doi:10.1186/s43141-023-00550-5)
Supplement: Supplementary file 2 — Additional file 2: Table S1. Accession numbers and GC content (%) of 18S, ITS and rbcL marker with the outgroups. Table S2. K2P genetic distance and disparity index (ID) between and within the Chlorellaceae genus. Table S3. Normalised Robinson Foulds(nRF) and Shimodaira Hasegawa test(SH test) on supermatrix marker datasets with alternative marker arrangements. [file 43141_2023_550_MOESM2_ESM.zip › Amended_Table_S3R1.docx]

| **Table S3**. Normalised Robinson Foulds (nRF) and Shimodaira Hasegawa test (SH test) on supermatrix marker datasets with alternative marker arrangements. | | | | | | | |
| --- | --- | --- | --- | --- | --- | --- | --- |
|  |  |  |  |  |  |  |  |
|  |  |  |  |  |  |  |  |
| (a) Marker | Tree (nRF = 22.5%) | |  |  |  |  |  |
|  | 18S-ITS | ITS-18S |  |  |  |  |  |
|  | p-value | |  |  |  |  |  |
| 18S-ITS | 0.77 | 0.925 |  |  |  |  |  |
| ITS-18S | 0.774 | 0.923 |  |  |  |  |  |
|  |  |  |  |  |  |  |  |
| (b) Marker | Tree (nRF = 7.5%) | |  |  |  |  |  |
|  | 18S-*rbcL* | *rbcL*-18S |  |  |  |  |  |
|  | p-value | |  |  |  |  |  |
| 18S-*rbcL* | 0.843 | 0.816 |  |  |  |  |  |
| *rbcL*-18S | 0.843 | 0.819 |  |  |  |  |  |
|  |  |  |  |  |  |  |  |
| (c) Marker | Tree (nRF = 2.5%) | |  |  |  |  |  |
|  | ITS-*rbcL* | *rbcL*-ITS |  |  |  |  |  |
|  | p-value | |  |  |  |  |  |
| ITS-*rbcL* | 0.96 | 0.953 |  |  |  |  |  |
| *rbcL*-ITS | 0.962 | 0.959 |  |  |  |  |  |
|  |  |  |  |  |  |  |  |
| (d) |  | Tree | | | | | |
|  |  | 18S-ITS-*rbcL* | 18S-*rbcL*-ITS | ITS-18S-*rbcL* | ITS-*rbcL*-18S | *rbcL*-18S-ITS | *rbcL*-ITS-18S |
| Tree | 18S-ITS-*rbcL* | 0.00% | 5.00% | 7.50% | 5.00% | 5.00% | 0.00% |
|  | 18S-*rbcL*-ITS | 5.00% | 0.00% | 7.50% | 5.00% | 2.50% | 5.00% |
|  | ITS-18S-*rbcL* | 7.50% | 7.50% | 0.00% | 5.00% | 7.50% | 7.50% |
|  | ITS-*rbcL*-18S | 5.00% | 5.00% | 5.00% | 0.00% | 5.00% | 5.00% |
|  | *rbcL*-18S-ITS | 5.00% | 2.50% | 7.50% | 5.00% | 0.00% | 5.00% |
|  | *rbcL*-ITS-18S | 0.00% | 5.00% | 7.50% | 5.00% | 5.00% | 0.00% |
| Marker | 18S-ITS-*rbcL* | 0.917 | 0.916 | 0.996 | 0.916 | 0.917 | 0.916 |
|  | 18S-*rbcL*-ITS | 0.916 | 0.916 | 0.995 | 0.915 | 0.915 | 0.916 |
|  | ITS-18S-*rbcL* | 0.917 | 0.917 | 0.995 | 0.916 | 0.916 | 0.916 |
|  | ITS-*rbcL*-18S | 0.912 | 0.912 | 0.995 | 0.912 | 0.911 | 0.912 |
|  | *rbcL*-18S-ITS | 0.921 | 0.92 | 0.995 | 0.921 | 0.92 | 0.92 |
|  | *rbcL*-ITS-18S | 0.916 | 0.915 | 0.996 | 0.914 | 0.915 | 0.915 |

Footnote: The bipartition differences between the supermatrix trees with alternate marker arrangement and the SH test (*P* value) for the congruency assessment between (a) 18S-ITS; (b) 18S-*rbcL*; (c) ITS-*rbcL*; (d) ITS-18S-*rbcL* and their corresponding alternative supermatrix arrangement.
